# Diaminopimelic Acid Metabolism by *Pseudomonadota* in the Ocean

**DOI:** 10.1128/spectrum.00691-22

**Published:** 2022-08-30

**Authors:** Li-Yuan Zheng, Ning-Hua Liu, Shuai Zhong, Yang Yu, Xi-Ying Zhang, Qi-Long Qin, Xiao-Yan Song, Yu-Zhong Zhang, Huihui Fu, Min Wang, Andrew McMinn, Xiu-Lan Chen, Ping-Yi Li

**Affiliations:** a State Key Laboratory of Microbial Technology, and Marine Biotechnology Research Center, Shandong University, Qingdao, China; b College of Marine Life Sciences, and Frontiers Science Center for Deep Ocean Multispheres and Earth System, Ocean University of China, Qingdao, China; c Laboratory for Marine Biology and Biotechnology, Pilot National Laboratory for Marine Science and Technology, Qingdao, China; d Institute for Marine and Antarctic Studies, University of Tasmania, Hobart, Tasmania, Australia; State Key Laboratory of Microbial Resources, Institute of Microbiology, Chinese Academy of Sciences

**Keywords:** diaminopimelic acid content, diaminopimelic acid decarboxylase, *Pseudomonadota*, seawater

## Abstract

Diaminopimelic acid (DAP) is a unique component of the cell wall of Gram-negative bacteria. It is also an important component of organic matter and is widely utilized by microbes in the world’s oceans. However, neither DAP concentrations nor marine DAP-utilizing microbes have been investigated. Here, DAP concentrations in seawater were measured and the diversity of marine DAP-utilizing bacteria and the mechanisms for their DAP metabolism were investigated. Free DAP concentrations in seawater, from surface to a 5,000 m depth, were found to be between 0.61 μM and 0.96 μM in the western Pacific Ocean. DAP-utilizing bacteria from 20 families in 4 phyla were recovered from the western Pacific seawater and 14 strains were further isolated, in which *Pseudomonadota* bacteria were dominant. Based on genomic and transcriptomic analyses combined with gene deletion and *in vitro* activity detection, DAP decarboxylase (LysA), which catalyzes the decarboxylation of DAP to form lysine, was found to be a key and specific enzyme involved in DAP metabolism in the isolated *Pseudomonadota* strains. Interrogation of the *Tara* Oceans database found that most LysA-like sequences (92%) are from *Pseudomonadota*, which are widely distributed in multiple habitats. This study provides an insight into DAP metabolism by marine bacteria in the ocean and contributes to our understanding of the mineralization and recycling of DAP by marine bacteria.

**IMPORTANCE** DAP is a unique component of peptidoglycan in Gram-negative bacterial cell walls. Due to the large number of marine Gram-negative bacteria, DAP is an important component of marine organic matter. However, it remains unclear how DAP is metabolized by marine microbes. This study investigated marine DAP-utilizing bacteria by cultivation and bioinformational analysis and examined the mechanism of DAP metabolism used by marine bacteria. The results demonstrate that *Pseudomonadota* bacteria are likely to be an important DAP-utilizing group in the ocean and that DAP decarboxylase is a key enzyme involved in DAP metabolism. This study also sheds light on the mineralization and recycling of DAP driven by bacteria.

## INTRODUCTION

Diaminopimelic acid (DAP), the ε-carboxyl derivative of lysine (Lys), is an essential component of the peptidoglycan of most Gram-negative bacteria (e.g., Escherichia, *Thalassospira*, and Pseudomonas) and of some Gram-positive bacteria (e.g., *Mycobacteria* and *Bacillus*) (1–3). Bacterial peptidoglycan is also an important refractory component of marine organic matter (4, 5). In the ocean, amino acids exist in two forms, free and combined (6, 7). While a large amount of marine DAP may exist in a combined form in marine bacterial peptidoglycan, free DAP is also present as a result of at least two types of microbial activity. First, DAP can be released when bacterial peptidoglycan debris is decomposed by extracellular enzymes secreted by marine microbes, such as proteases, resulting in the release of free DAP into seawater (4). Second, DAP can be synthesized from aspartic semialdehyde and pyruvate by some bacteria (8); the synthesized DAP is released into the seawater when these bacteria are lysed by viruses or predatory bacteria (9–11). However, little attention has been paid to either the concentration or the metabolism of marine DAP. It is still unknown which bacteria catabolize free DAP and contribute to the mineralization and recycling of DAP in the ocean.

Although no marine DAP-utilizing bacteria have been reported so far, the occurrence of DAP in terrestrial pathogenic bacteria has drawn much attention. DAP is an intermediate metabolite of intracellular Lys synthesis in bacteria. DAP-related enzymes in this pathway, which are absent from mammals (12–14), provide targets for drug development. DAP decarboxylase catalyzes the decarboxylation of DAP to form Lys in bacterial Lys biosynthesis. The amino acid sequence of DAP decarboxylase was first reported in Bacillus methanolicus in 1993 (15). Furthermore, the crystal structures of several DAP decarboxylases have been solved, including nine from terrestrial bacteria and two from the marine thermophilic bacteria Thermotoga maritima and Methanocaldococcus jannaschii (16, 17). However, it is unknown whether marine DAP-utilizing bacteria metabolize DAP with DAP decarboxylase.

The aim of this study was to investigate both the diversity of DAP-utilizing bacteria in seawater and the mechanism(s) of their DAP metabolism. Sea water samples were collected from several sites and depths in the western Pacific Ocean. After the DAP concentrations in the seawater samples were measured, the diversity of the DAP-utilizing bacteria in the samples was analyzed by enrichment culture, and 14 DAP-utilizing bacterial strains were isolated. Based on genomic and transcriptomic analyses, combined with gene deletion and *in vitro* activity detection, DAP decarboxylase was identified as a key and specific enzyme involved in DAP metabolism in the isolated strains. Based on a search of the homologs of DAP decarboxylase in the *Tara* Oceans database (http://ocean-microbiome.embl.de/companion.html), the distribution and diversity of DAP-utilizing bacteria in the global oceans were predicted. The results provide an insight into DAP metabolism by marine bacteria.

## RESULTS

### Determination of the concentrations of free DAP in the seawater samples.

To determine the concentrations of free DAP in the western Pacific Ocean water samples, a standard curve of DAP concentrations was plotted based on detection by FDAA [*N*^α^-(2, 4-dinitro-5-fluorophenyl)-l-alaninamide]-derived high-pressure liquid chromatography (HPLC) (see Fig. S1 in the supplemental material). Because the concentration of free DAP in the seawater was too low to be detected by FDAA-derived HPLC, the sample volumes were concentrated (1:5). Then, the free DAP in the concentrated samples was measured by FDAA-derived HPLC (Fig. S2), and the DAP concentration in each sample was calculated based on the standard curve. DAP concentrations in the seawater samples from 0, 1,000, 3,000, and 5,000 m were 0.61 ± 0.02, 0.83 ± 0.05, 0.96 ± 0.01, and 0.93 ± 0.01 μM, respectively. The concentrations (0.83 μM to 0.96 μM) in the three deep-sea samples from 1,000 m, 3,000 m, and 5,000 m were similar, but were much higher than that in the surface sample (0.61 μM).

### Diversity of DAP-utilizing bacteria in western Pacific seawater.

**FIG 1:**
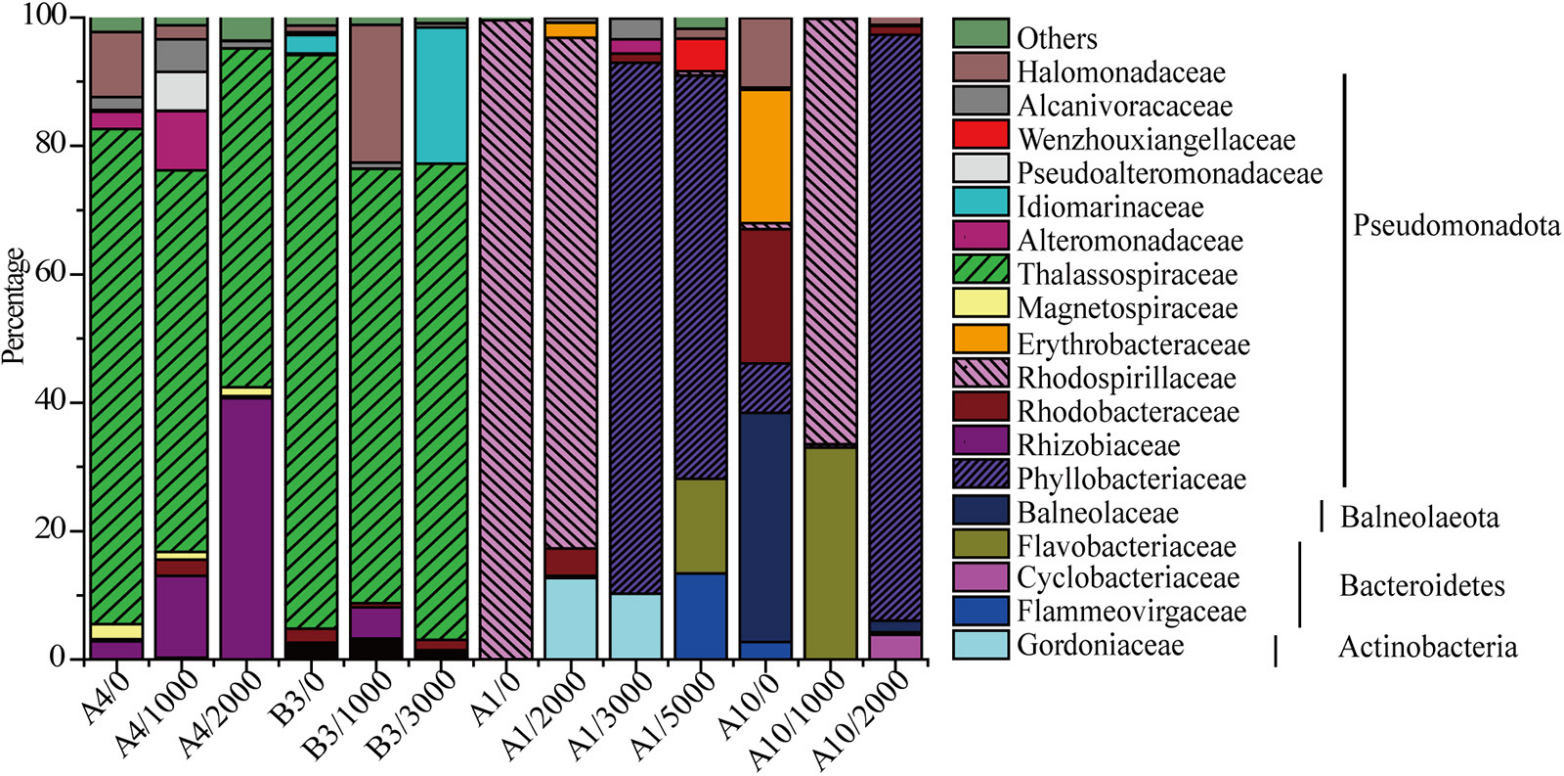
Taxonomic composition of DAP-utilizing bacteria recovered from western Pacific Ocean seawater samples. Bacteria were recovered by enrichment culture with 5 mM DAP as the sole carbon and N source. Seawater samples were collected from different depths at stations in the western Pacific (Table S1). The numbers after the slashes in the sample names are the water depths (in meters) of the samples.

To investigate the diversity of DAP-utilizing bacteria in the western Pacific Ocean, bacteria were recovered from 13 samples from 4 stations by enrichment culture in a medium containing DAP as the sole carbon and nitrogen (N) source (Table S1). After 4 days of culture at 20°C, the microbial community composition of each sample was analyzed using the 16S rRNA gene sequences (Fig. 1). A total of 20 families, falling into four phyla, were detected; one family (*Gordoniaceae*) in *Actinobacteria*, one family (*Balneolaceae*) in *Balneolaeota*, three families (*Cyclobacteriaceae*, *Flammeovirgaceae*, and *Flavobacteriaceae*) in *Bacteroidetes*, and 13 families (*Alteromonadaceae*, *Alcanivoracaceae*, *Erythrobacteraceae*, *Halomonadaceae*, *Idiomarinaceae*, *Magnetospiraceae*, *Phyllobacteriaceae*, *Pseudoalteromonadaceae*, *Rhizobiaceae*, *Rhodobacteraceae*, *Rhodospirillaceae*, *Thalassospiraceae*, and *Wenzhouxiangellaceae*) in *Pseudomonadota*. Thus, *Pseudomonadota* was the most prevalent phylum. Nevertheless, community composition at the family level differed between stations (Fig. 1). *Thalassospiraceae* was dominant in stations A4 (0 m, 77%; 1,000 m, 59%; 2,000 m, 53%) and B3 (0 m, 89%; 1,000 m, 68%; 3,000 m, 74%), *Rhodospirillaceae* dominated in A1 (0 m, 99%; 2,000 m, 79%) and A10 (1,000 m, 94%), and *Phyllobacteriaceae* was most abundant in A1 (3,000 m, 83%; 5,000 m, 63%) and A10 (2,000 m, 91%). *Thalassospiraceae* abundance was indirectly proportional to depth, while *Phyllobacteriaceae* showed the opposite trend (Fig. 1). Together, the results indicate a high diversity of DAP-utilizing bacteria in western Pacific seawater, suggesting that a wide range of marine bacteria may have a DAP-utilizing ability.

### Isolation of DAP-utilizing bacteria and analysis of their DAP-utilizing ability.

Fourteen DAP-utilizing bacterial strains were further isolated from the enrichment culture on screening plates with DAP as the sole N source (Table 1). Of these, one strain was from the family *Microbacteriaceae* (phylum *Actinobacteria*) and one was from the family *Flavobacteriaceae* (phylum *Bacteroidetes*), while the remaining 12 were from the phylum *Pseudomonadota*. Of the 12 *Pseudomonadota* strains, eight were affiliated with the families *Erythrobacteraceae*, *Rhodobacteraceae*, and *Rhodospirillaceae* (*Alphaproteobacteria*) and four with the families *Halomonadaceae*, *Pseudoalteromonadaceae*, and *Alteromonadaceae* (*Gammaproteobacteria*).

**TABLE 1:** Strains isolated with DAP as the sole N source

Station	Depth(s) (m)	Strain	Top-hit strain	Sequence lengths^*a*^ (% 16S rRNA gene identity)
A3	0; 1,000	A30-3	Erythrobacter citreus MTFD734	1,441/1,450 (99)
A3	2,000	A32-1	Microbacterium saccharophilum K-1	1,498/1,512 (99)
A4	0	A40-3	Thalassospira tepidiphila P1-D1	1,469/1,474 (99)
A4	0	A40-4	Halomonas janggokensis MT0-7-1	1,474/1,476 (99)
A4	1,000	A41-2	Pseudoalteromonas donghaensis HJ51	1,536/1,538 (99)
A4	1,000	A41-4	Alteromonas mediterranea UM8	1,356/1,357 (99)
A4	2,000	A42-5	Labrenzia aggregata CECT 4801	1,325/1,325 (100)
B3	0	B30-1	Thalassospira xiamenensis M-5	1,489/1,490 (99)
B3	0	B30-2	*Sulfitobacter* sp. strain SK025	1,456/1,456 (100)
B3	0	B30-3	*Thalassospira* sp. strain 4G	1,312/1,312 (100)
B3	0	B30-5	Zunongwangia profunda MT2-7	1,385/1,387 (99)
B3	1,000	B31-1	Pseudooceanicola nanhaiensis MTd1-11	1,383/1,383 (100)
B3	1,000	B31-4	Thalassospira permensis Mix10	1,287/1,289 (99)
B3	1,000	B31-7	Alteromonas australica MTg9-7	1,518/1,530 (99)

a Given as query sequence length/alignment sequence length, in base pairs.

**FIG 2:**
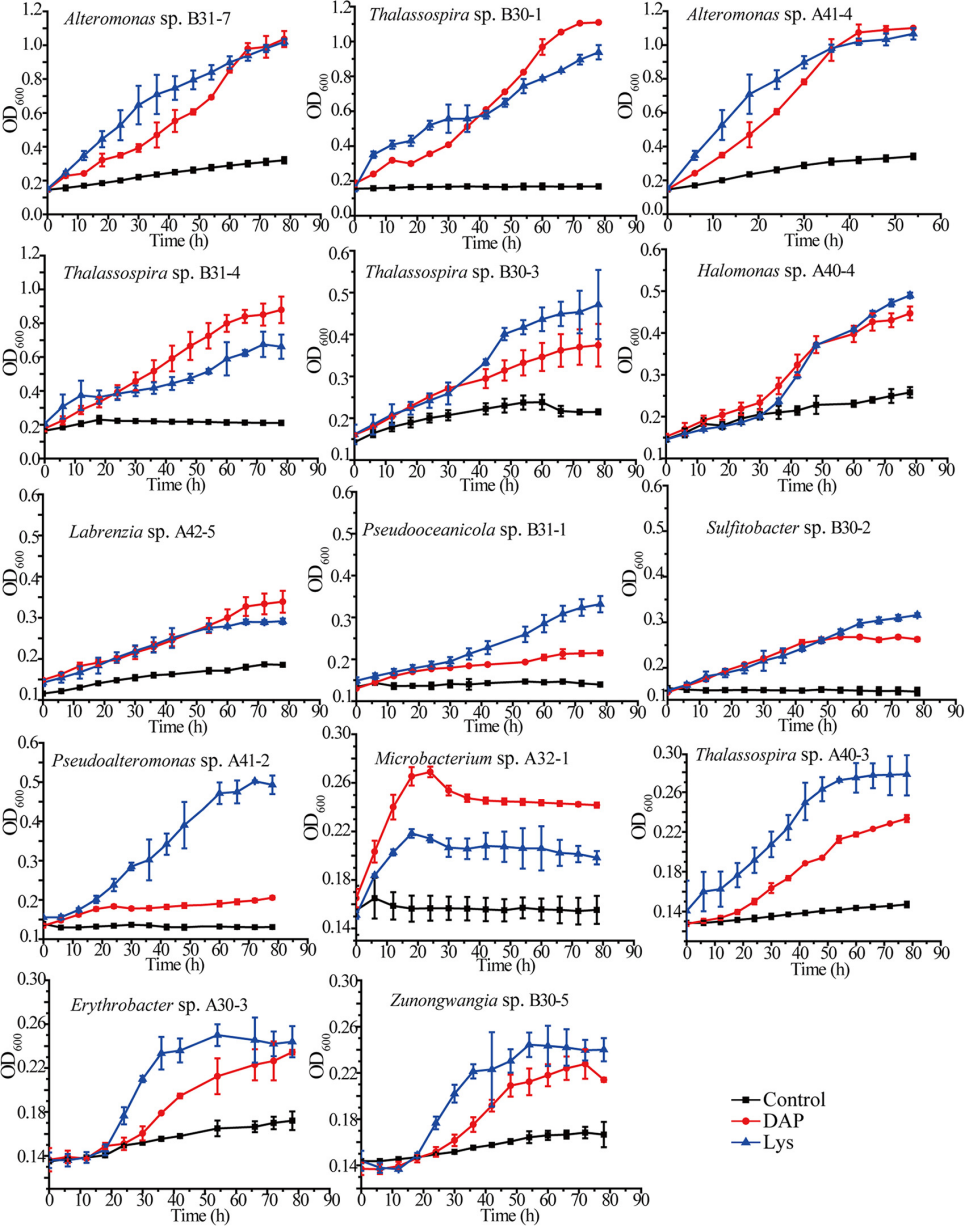
Growth curves of the 14 isolated strains cultured with DAP or Lys as the sole N source. Strains were cultured at 25°C and 180 rpm. The growth curves of the strains cultured in the Ndap medium (DAP), in the same medium with Lys instead of DAP (Lys), and in the same medium without DAP (control) are presented. The error bars represent standard deviations from triplicate experiments.

To further confirm the ability of the isolated bacterial strains to utilize DAP, the growth rates of these strains were measured in Ndap liquid medium containing 5 mM DAP as the sole N source. The growth rates of the strains cultured in the same medium with Lys instead of DAP were used as a control. As shown in Fig. 2, all strains showed significant growth in Ndap medium despite differences in their growth rates, which indicates that all strains have the ability to utilize DAP as a N source.

### Identification of the key gene *lysA* involved in DAP metabolism.

**FIG 3:**
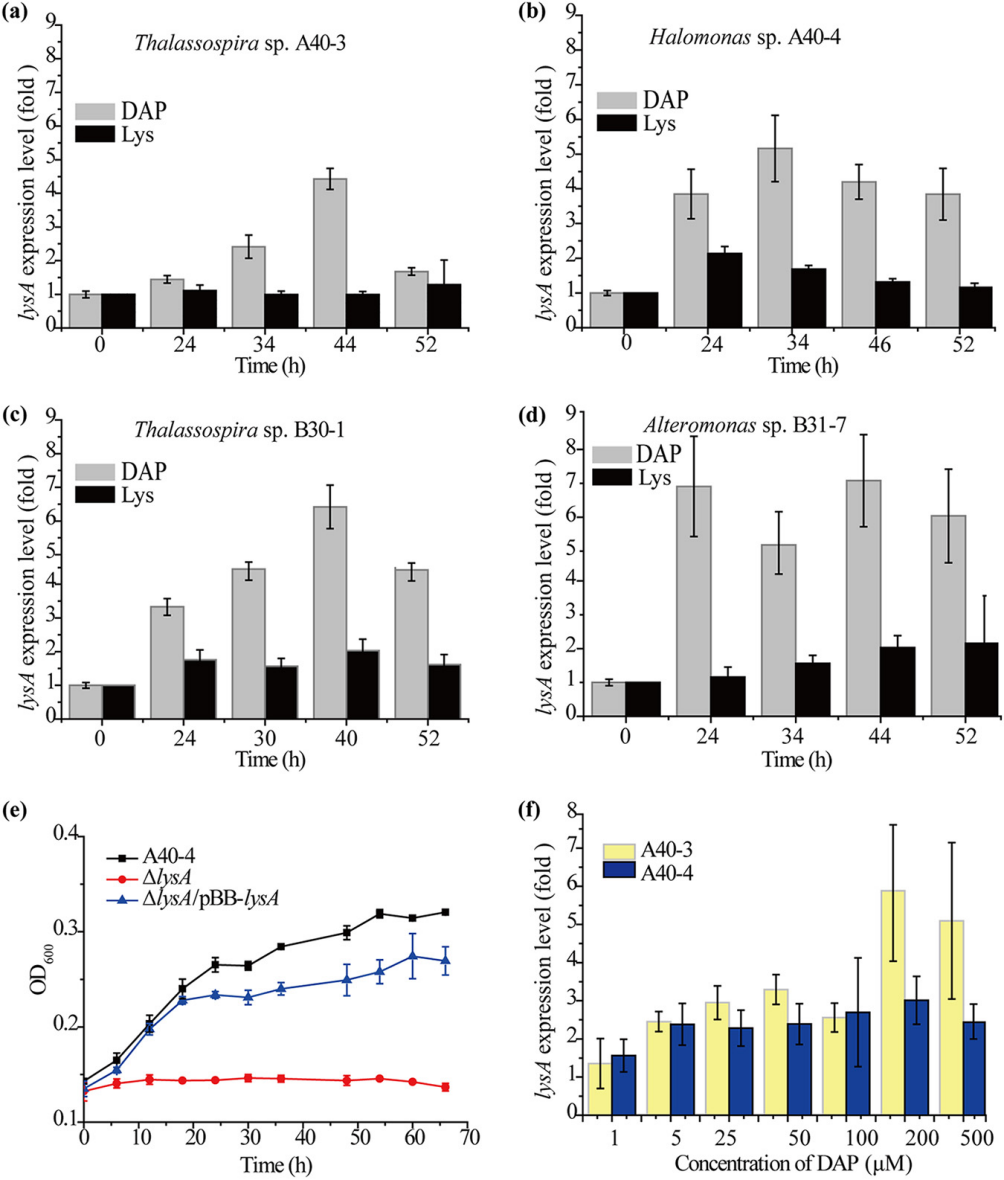
Analyses of the transcription and function of gene *lysA* in DAP-utilizing bacteria. (a to d) Relative transcriptional levels of *lysA* in strains A40-3 (a), A40-4 (b), B30-1 (c), and B31-7 (d) detected by RT-qPCR. Bacteria were cultured at 25°C and 180 rpm with 5 mM DAP or lysine as the sole N source. (e) Growth curves of strain A40-4, its *lysA* deletion (Δ*lysA*) mutant, and the complemented Δ*lysA*/pBB*lysA* strain. A40-4 and the Δ*lysA* and Δ*lysA*/pBB*lysA* mutants were cultured at 25°C and 180 rpm in Ndap medium. (f) Induction effects of different concentrations of DAP on the transcription of *lysA* in strains A40-3 and A40-4. Bacteria were cultured in a medium containing different concentrations of DAP and 3% sea salt (Sigma, USA) at 25°C for 12 h. The error bars represent standard deviations from triplicate experiments.

Based on the above results of the enrichment culture and diversity analyses (Fig. 1), as well as isolation (Table 1) and DAP-utilizing ability analysis (Fig. 2), *Thalassospira* was found to likely be an important group for marine DAP metabolism. Therefore, a *Thalassospira* strain, A40-3, was selected to identify the key gene(s) involved in DAP metabolism via genomic and transcriptomic analyses, noting that the clear differences in the growth of this strain between DAP and Lys may favor comparative transcriptome analysis. When strain A40-3 was cultured with DAP to the middle of the logarithmic growth phase, 229 genes were upregulated compared with the 0-h sample; of these, 71 encoded putative enzymes based on functional annotation (Fig. S3). Furthermore, among these enzyme genes, 12 were significantly upregulated on DAP but not on Lys (Table 2). Of these, the gene *lysA* (IT893_16705) (Fig. S4) was predicted to encode a DAP decarboxylase (Table S2) that catalyzes the conversion of DAP to Lys (11), which therefore may be directly involved in DAP metabolism. The other 11 genes were not further investigated in this study because they were not closely related to DAP metabolism, based on the gene function annotation. Based on the transcriptomic analysis, *lysA* was significantly upregulated in strain A40-3 when it was cultured with DAP (fold change, 6.11; *P* < 0.01) but not when it was cultured with Lys (fold change, 0.75; *P* < 0.01) (Table 2); this was further supported by the reverse transcription quantitative real-time PCR (RT-qPCR) analysis (Fig. 3a; Fig. S5).

**TABLE 2:** Enzyme genes upregulated on DAP but not on Lys in *Thalassospira* sp. strain A40-3^*a*^

Gene (protein)	DAP		Lysine	
	Fold change^*b*^	*P* value^*c*^	Fold change^*b*^	*P* value^*c*^
*carB* (carbamoyl-phosphate synthase)	8.66	1.66E−08	−1.19	3.29E−15
*metB* (*O*-succinylhomoserine sulfhydrylase)	7.78	7.39E−15	−1.97	2.52E−52
*thrA* (aspartokinase)	6.37	2.65E−265	−0.61	5.02E−04
*lysA* (diaminopimelate decarboxylase)	6.11	1.01E−092	0.75	2.08E−07
*aroA* (cytidylate kinase)	5.30	1.13E−07	−0.84	4.31E−06
*aro* (B3-dehydroquinate synthase)	5.13	2.86E−07	0.62	3.18E−06
*hisA* (imidazole-4-carboxamide isomerase)	5.04	4.68E−07	0.82	2.91E−05
*pyc* (pyruvate carboxylase)	2.95	2.35E−51	−3.93	9.44E−181
*fumA* (fumarate hydratase class I)	2.56	4.00E−42	−3.88	6.62E−138
*glyA* (serine hydroxymethyltransferase)	2.31	1.39E−27	0.36	7.17E−03
*HPD* (hemolysin)	2.19	2.20E−03	−5.98	4.27E−190
*glcD* (glycolate oxidase subunit)	2.09	1.78E−27	0.66	8.52E−06

a The resting cells of the strain were cultured with DAP or Lys as the sole N source to the middle of the logarithmic growth phase, and then cells were collected as the DAP sample and the Lys sample. The resting cells in sea salt solution were taken as the 0-h sample.

b Calculated by comparing the transcription level of the gene in the DAP or Lys sample to that in the 0-h sample.

c Calculated by Fisher’s exact test with FDR correction.

To investigate the universality of the gene *lysA*, the draft genomes of three other strains (A40-4, B30-1, and B31-7) from different genera and with noticeable DAP-utilizing ability were obtained, and *lysA* was found to be present (Table S2). As when strain A40-3 was cultured with 5 mM DAP, the transcription levels of the *lysA* genes were all significantly upregulated during growth, especially in the exponential phase. However, they were not upregulated when Lys was used as a substrate (Fig. 3b to d). This suggests that *lysA* may be a key enzyme gene involved in DAP metabolism in marine DAP-utilizing bacteria. To test this, an attempt was made to remove *lysA* from these four strains (A40-3, A40-4, B30-1, and B31-7), but this was successful only in strain A40-4. The Δ*lysA* strain, a *lysA* deletion mutant of strain A40-4, and the complemented Δ*lysA*/pBB*lysA* strain were constructed. The Δ*lysA* strain showed almost no growth in the culture with DAP, indicating that it had almost completely lost the ability to utilize DAP (Fig. 3e). The Δ*lysA*/pBB*lysA* strain showed significant growth compared to the Δ*lysA* strain, indicating that its ability to utilize DAP had been restored (Fig. 3e). This result indicates that *lysA* was essential for DAP metabolism in strain A40-4 when DAP was utilized as a N source.

To determine the lowest concentration of DAP that induces the transcription of *lysA* in marine bacteria, the inducing effects of different concentrations of DAP on the transcription of *lysA* in strains A40-3 and A40-4 were investigated. The results show that, while 1 μM DAP in the medium could induce the transcription of *lysA* above background levels, 5 μM DAP significantly induced its transcription in both strains (Fig. 3f).

### Functional analysis of the *lysA* genes *in vitro*.

**FIG 4:**
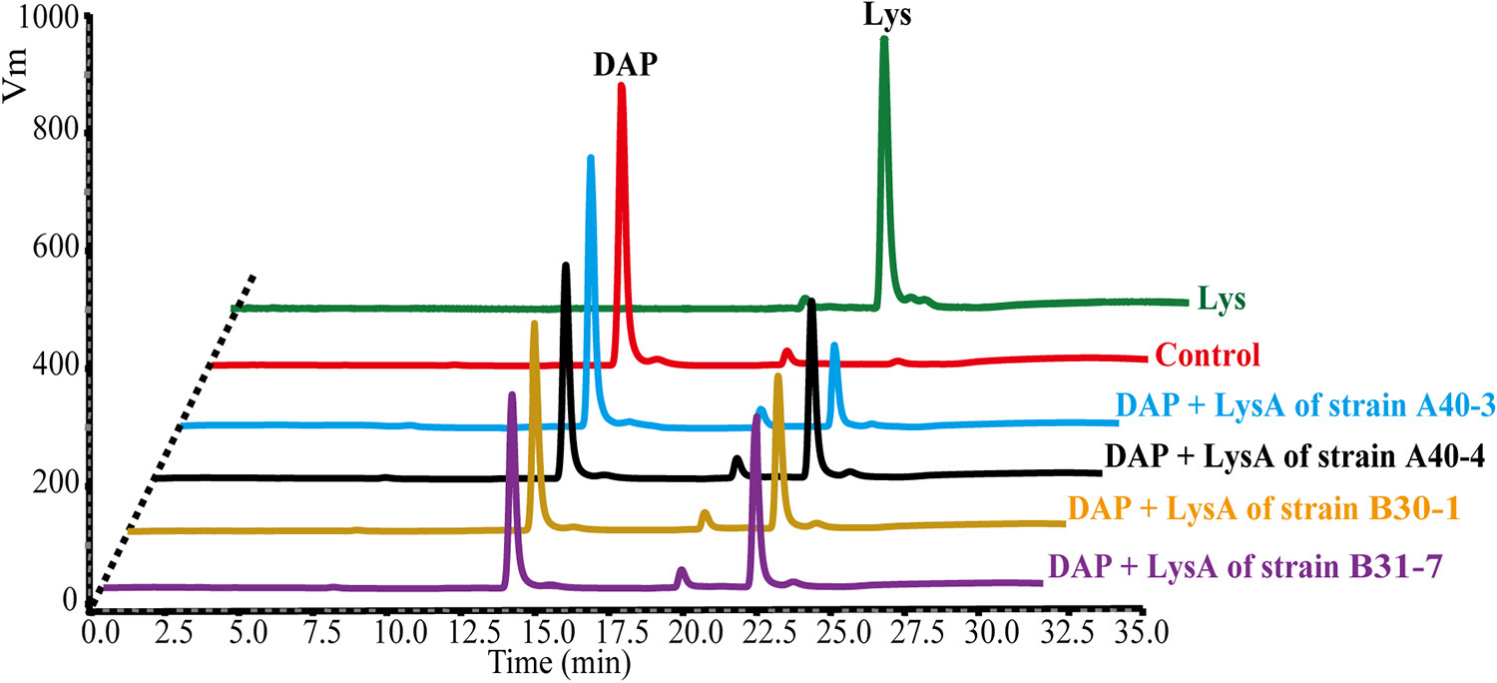
Detection of the DAP decarboxylase activities of the recombinant LysA proteins from strains A40-3, A40-4, B30-1, and B31-7. Recombinant LysA proteins were mixed with 200 mM Tris-HCl (pH 8.0), 100 mM PLP, and 5 mM DAP. The mixtures were incubated at 25°C for 10 min, and the reactions were stopped by the addition of 10% TFA to the mixtures. The concentrations of DAP and Lys in the reaction mixtures were determined with an automatic amino acid analyzer (L-8900; Hitachi). The control assay had the same reaction system except that the LysA protein was not added. The graph shows representative results of triplicate experiments.

The *lysA* genes in the marine strains were predicted to encode DAP decarboxylase enzymes that could catalyze the decarboxylation of DAP to form Lys. To confirm this, the *lysA* genes from strains A40-3, A40-4, B30-1, and B31-7 were expressed in Escherichia coli BL21(DE3). The recombinant LysA proteins were purified, and their activity toward DAP was detected by monitoring the production of Lys using an automatic amino acid analyzer (Fig. 4). The four LysA proteins were all able to convert DAP to Lys, demonstrating that they were all DAP decarboxylases. The specific activities of these enzymes ranged from 12.68 U mg^−1^ to 32.77 U mg^−1^ (Table 3). Their *K_m_* values for DAP ranged from 0.72 mM to 1.90 mM (Table 3; Fig. S6). These results, together with the above results obtained with *lysA*, suggest that marine bacteria metabolize DAP to Lys by DAP decarboxylase when they utilize DAP as a N source.

**TABLE 3:** Specific enzyme activity and kinetic parameters of the recombinant LysA proteins (DAP decarboxylases) for DAP

Strain	Sp act (U mg^−1^)	*K_m_* (mM)^*a*^	*k*_cat_ (s^−1^)	*V*_max_ (U mg^−1^)
A40-4	29.87 ± 2.31	0.72 ± 0.14	41.63 ± 0.88	58.28 ± 1.24
A40-3	12.68 ± 0.38	1.89 ± 0.34	26.94 ± 2.42	37.71 ± 3.38
B30-1	32.77 ± 0.62	1.17 ± 0.34	40.33 ± 3.73	46.79 ± 4.33
B31-7	15.34 ± 0.73	0.99 ± 0.24	13.38 ± 2.49	18.33 ± 3.41

a Kinetic parameters were calculated by nonlinear regression fitted directly to the Michaelis-Menten equation using Origin8 software. The initial rates were determined with 0 to 12 mM DAP. The nonlinear fit curves for the decarboxylation of DAP by LysA proteins are shown in Fig. S6.

### Analysis of the mechanism of DAP metabolism by marine bacteria.

When DAP is the sole N source, marine DAP-utilizing bacteria need to convert DAP into l-amino acids for protein synthesis. The above results show that they can use the enzyme DAP decarboxylase to convert DAP to Lys for protein synthesis and cell growth. DAP might also be involved in DAP-type peptidoglycan synthesis for cell wall construction via the enzymes MurE (UDP-*N*-acetylmuramoyl-l-alanyl-d-glutamate-2,6-diaminopimelate ligase) and MurF (UDP-*N*-acetylmuramoyl-tripeptide-d-alanyl-d-alanine ligase), which have been reported to be involved in DAP incorporation into bacterial DAP-type peptidoglycan synthesis (18–21). The RT-qPCR analysis also supported this suggestion. The transcription levels of both the *murE* and *murF* genes were significantly upregulated with either DAP or Lys (Fig. S7) in all 4 cultured strains, suggesting that *murE* and *murF*, despite not being DAP specific, are likely to be involved in peptidoglycan biosynthesis in DAP-utilizing bacteria.

### Diversity and distribution of marine bacteria containing the *lysA* gene.

**FIG 5:**
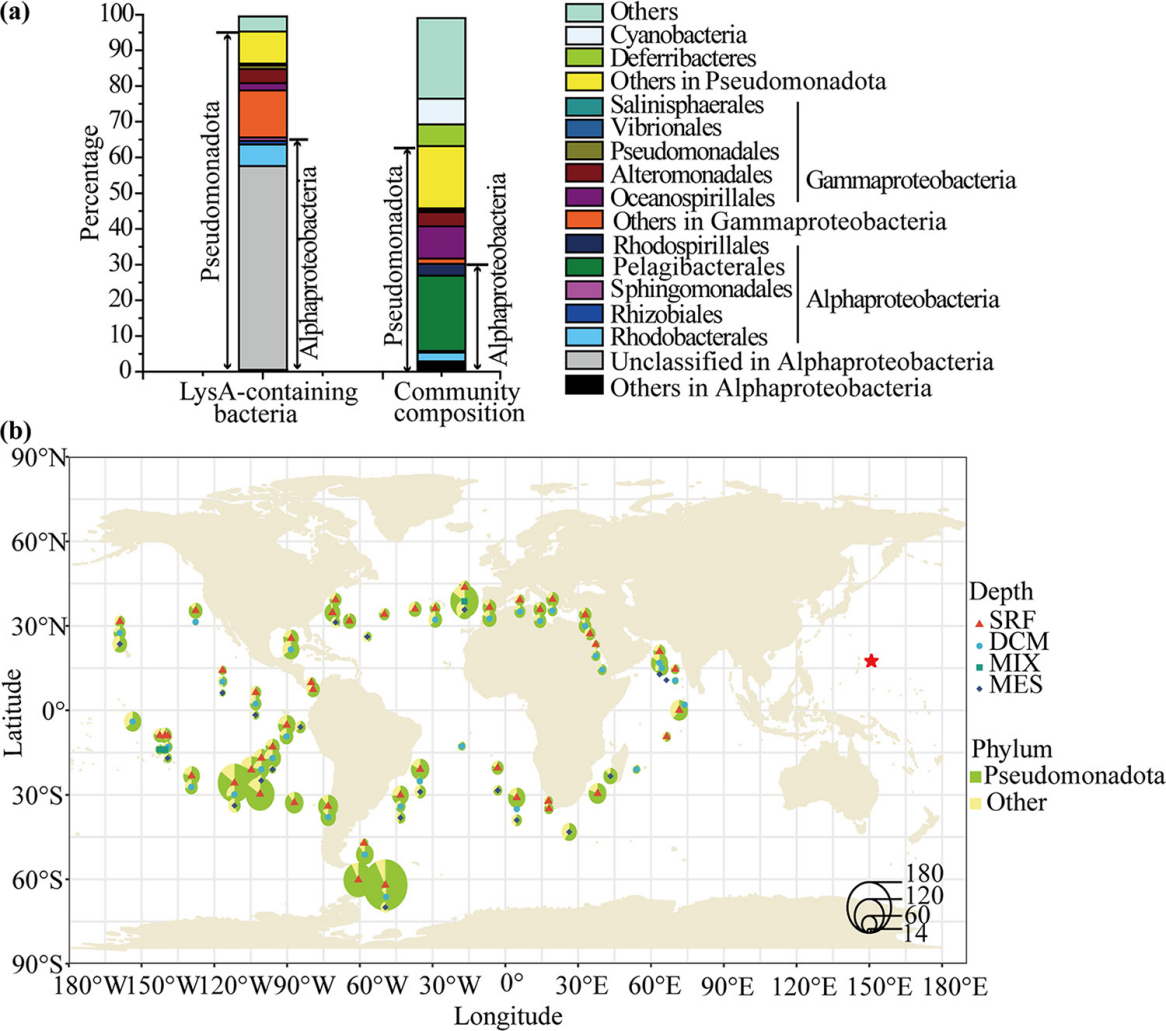
Taxonomic composition and distribution of marine bacteria containing the *lysA* gene in the *Tara* Oceans database. (a) Taxonomic compositions of bacteria containing the *lysA* gene and the microbial community retrieved from *Tara* Oceans database. *lysA* sequences were obtained from the *Tara* Oceans database (http://ocean-microbiome.embl.de/companion.html) based on a tBLASTn search using the LysA sequence from strain A40-3 with an E-value cutoff of 1E−30. The taxonomic composition of the microbial community was analyzed based on 16S rRNA gene sequences derived from the *Tara* Oceans database. The proportion of each taxon in the bacteria containing the *lysA* gene or in the microbial community is indicated by its percentage. (b) Distribution of bacteria containing the *lysA* gene in the ocean. The numbers of unique hits of *lysA* sequences were normalized to the numbers of unique *recA* sequences to estimate the abundance of *lysA* in the *Tara* Oceans database. The sizes of the pie charts represent the numbers of *lysA* sequences, and the green area in each pie chart indicates the proportion of *lysA* sequences from *Pseudomonadota* (Wilcoxon test *P* value < 0.001). Samples in surface water layer (SRF), deep chlorophyll maximum layer (DCM), subsurface epipelagic mixed layer (MIX), and mesopelagic zone (MESO) were analyzed. The sampling location of this study is marked with a red star, and detailed information on sampling sites is shown in Table S1 and Fig. S8.

The diversity and distribution of *lysA*-containing marine microbes were further investigated by searching the *Tara* Oceans database with the LysA sequence of *Thalassospira* sp. strain A40-3 (Fig. S4) as a blast query. A total of 8,311 *lysA* sequences out of 40,154,823 sequences were detected, of which 7,300 *lysA* sequences were unique. The taxonomic composition of bacteria containing *lysA* sequences and that of the microbial community retrieved from the *Tara* Oceans database are shown in Fig. 5a. The detected *lysA* sequences were mainly found in the phylum *Pseudomonadota*, accounting for 92%. The proportion of *Pseudomonadota* in the microbial community of the *Tara* Oceans database was 65.1%. At the class level, *Alphaproteobacteria* was the most abundant group containing *lysA* sequences, accounting for 65%. The proportion of *Alphaproteobacteria* in the microbial community was only 30.2%. In *Alphaproteobacteria*, most *lysA* sequences were from unclassified bacteria (56.6%) and the order *Rhodobacterales* (6%). However, unclassified bacteria and *Rhodobacterales* accounted for only 0.002% and 2.5%, respectively, of the microbial community. *Gammaproteobacteria* was the second most abundant class containing *lysA* sequences, accounting for 18.0%. The proportion of *Gammaproteobacteria* in the microbial community in the *Tara* Oceans database was 16.7%. In *Gammaproteobacteria*, *lysA* sequences were mainly distributed in the orders *Alteromonadales* (4.0%), *Oceanospirillales* (2.0%) and *Pseudomonadales* (1.0%). Thus, based on the data from the *Tara* Oceans database, marine bacteria containing *lysA* sequences were mainly from *Pseudomonadota*.

The distribution of *lysA-*containing bacteria in the global marine environments was further investigated by searching the *Tara* Oceans database (Fig. 5b). The results show that bacteria containing *lysA* sequences were present at 68 sampling stations in the *Tara* Oceans database, which were widely distributed in the global oceans. In addition, the upper ocean, including the surface water layer, deep chlorophyll maximum layer, and subsurface epipelagic mixed layer, contained more *lysA* sequences than the mesopelagic ocean (Wilcoxon test *P* value < 0.001).

## DISCUSSION

**FIG 6:**
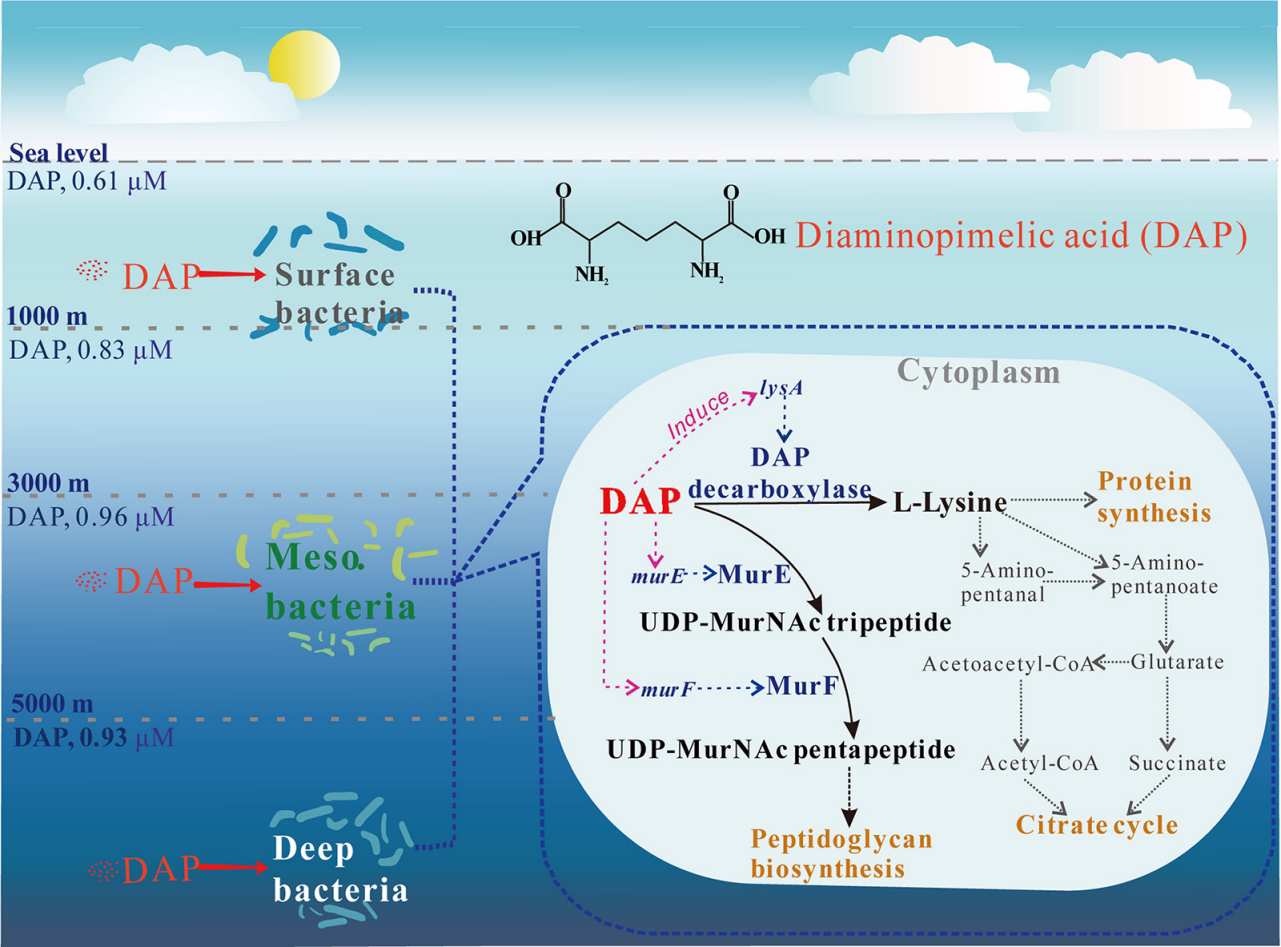
Model of bacterial DAP metabolism in the ocean. Key enzymes are in blue. The solid black arrows represent the reactions catalyzed by the key enzymes. DAP in the ocean can be utilized by marine bacteria at different water depths. When DAP is used as the N source, the expression of *lysA*, *murE*, and *murF* is induced. When transported into the cell of a marine bacterium, some DAP molecules are decarboxylated by DAP decarboxylase to Lys, which can be either directly used or eventually converted to acetyl-CoA or succinic acid and further metabolized by the tricarboxylic acid cycle to provide intermediates and energy for cell growth; in the meantime, some DAP molecules are incorporated into the peptide chain of the DAP-type peptidoglycan for cell wall construction via MurE and MurF.

The diversity of marine DAP-utilizing bacteria and the mechanism of their DAP metabolism have so far been little investigated. In this study, the diversity of marine DAP-utilizing bacteria in the western Pacific Ocean, from the surface to a 5,000 m depth, was investigated using an enrichment culture at 20°C with DAP as the sole carbon and N source, and 14 DAP-utilizing bacterial strains were further isolated from the enrichment culture. It is noticeable that the results from both the enrichment culture and the isolations showed that *Pseudomonadota* is the main bacterial group to utilize DAP in the western Pacific Ocean. However, it is also worth noticing that some DAP-utilizing bacteria may have been unrecoverable from the seawater samples due to the specifics of the enrichment and isolation culture conditions (including temperature, pressure, and others) and that the Ndap medium may favor LysA-carrying *Pseudomonadota*. Although the diversity of marine DAP-utilizing bacteria has never previously been reported, in recent years, several reports on the diversity of marine d-amino acid-utilizing bacteria, based on enrichment culture and isolation culture (22, 23), have been published; these studies also showed that *Pseudomonadota* (*Proteobacteria* in the literature) is the main bacterial group to utilize d-amino acids in the ocean.

d-Amino acids (DAA) are metabolized by marine bacteria through several pathways involving a variety of key enzymes, including DAA oxidoreductases/dehydrogenases, d-serine ammonia-lyases, d-serine ammonia-lyase DSD1s, and DAA transaminases, to convert DAAs into α-keto acids and amino acid racemases to convert DAAs into LAAs (22, 24–29). Different bacterial strains are able to use different enzymes to metabolize d-amino acids, and some d-amino acids can be metabolized by different enzymes in different bacterial strains (22). However, it was found here that the four investigated *Pseudomonadota* strains from different genera seemed to use the same pathway to utilize DAP. They used DAP decarboxylase as a key enzyme to convert DAP to Lys in DAP catabolism. While it is still possible that other DAP catabolism pathways are present in the ocean, this awaits further investigation. Our bioinformational investigation of the *Tara* Oceans database showed that bacteria carrying genes encoding DAP decarboxylase are widely distributed in the ocean. These bacteria potentially metabolize DAP through the mechanisms shown in the model presented in Fig. 6. When transported into the bacterial cell, some DAP molecules are decarboxylated by the DAP decarboxylase LysA to Lys, which is then either directly used for protein synthesis or converted to acetyl coenzyme A (acetyl-CoA) and succinic acid, which are further metabolized by the tricarboxylic acid cycle to provide intermediates and energy for cell growth (30). Some DAP molecules are also incorporated into the peptide chain of the DAP-type peptidoglycan for cell wall construction via the enzymes MurE, MurF, and others (18–20).

It has been reported that the concentration of total dissolved free amino acids (DFAA) in Atlantic Ocean surface water is 0.7 to 2.9 μM (31). However, DFAA concentrations in Pacific Ocean surface waters have been reported to be 40 to 50 nM (32). Several other studies have also suggested that free amino acids occur in the low nanomolar range in the surface ocean (33, 34). So far, however, the concentration or amount of DAP in the ocean remains unknown. In this study, it was found that the concentrations of free DAP from the surface to a depth of 5,000 m in the western Pacific Ocean ranged from 0.61 μM to 0.96 μM; those from 1,000 m to 5,000 m were present at a relatively constant high level (0.83 μM to 0.96 μM). However, the small volume of the samples and the concentration techniques applied may have influenced the accuracy of the detection. Other concentrating techniques, such as solid-phase extraction and tangential flow ultrafiltration, might be appropriate if larger sample volume is available (35–37). The concentrations of total hydrolysable amino acids (THAA), including DFAA and dissolved combined amino acids (DCAA), are often 10 times higher than those of DFAA in surface Pacific waters (32). Thus, due to the large amount of combined DAP in bacterial cell wall debris, the total content of DAP in both the combined and the free forms in seawater is likely to be much higher than those actually measured in this study. In the ocean, while free DAP is taken up by bacteria and metabolized for growth and for DAP-type peptidoglycan synthesis, free DAP is also released when bacterial DAP-type peptidoglycan is decomposed (38–41) by enzymes secreted by microbes. These microbial activities lead to a dynamic balance of DAP in seawater. We found that 1 μM extracellular DAP can induce the transcription of *lysA* above background levels in *Thalassospira* sp. strain A40-3 and *Halomonas* sp. strain A40-4. Although this concentration is a little higher (5.5% to 21.7%) than those measured in deep seawater, the transcription of *lysA* in DAP-utilizing bacteria is likely to have been induced by *in situ* seawater DAP, considering that the content of DAP *in situ* seawater fluctuates due to the microbial activities discussed above. In addition, the *K_m_* values (0.72 mM to 1.90 mM) of the four studied LysA proteins for DAP are much higher than the concentrations of DAP measured in seawater, implying that the DAP-utilizing bacteria may be able to absorb and accumulate DAP at a higher concentration in their cells before DAP is further metabolized by enzymes like LysA. This, however, awaits further investigation by a DAP labeling experiment.

In summary, this study investigated the diversity of DAP-utilizing bacteria in seawater by cultivation and bioinformational analysis, and the bacterial DAP metabolic mechanism by omics analysis combined with genetic and biochemical experiments. The results reveal that marine DAP is metabolized by heterotrophic bacteria, with *Pseudomonadota* as the main group, and that the DAP decarboxylase LysA is a key and specific enzyme involved in DAP metabolism in marine bacteria. This study provides an insight into DAP metabolism in the ocean, which contributes to our understanding of the mineralization and recycling of marine DAP driven by microbes.

## MATERIALS AND METHODS

### Sampling.

Thirteen seawater samples from different depths were collected with Niskin in-line water samplers from 5 stations (A1, A3, A4, A10, and B3) during typical seamount ecosystem research in the western Pacific Ocean in April 2018. The cruise was conducted on the research vessel Kexue. Samples (40 mL each) were stored in 50-mL aseptic bottles at 4°C for 30 days before being processed in the lab. Information on the sampling sites and depths is shown in Table S1 and Fig. S8.

### Determination of DAP concentrations in seawater samples.

Concentrations of DAP were determined by FDAA [*N*^α^-(2,4-dinitro-5-fluorophenyl)-l-alaninamide; Sigma, USA] derivatization and HPLC as described by Dołowy and Pyka (42). Briefly, the samples (1 mL) (Table S1) were concentrated to 0.2 mL at 4,000 × *g* for 3 to 6 h at 45°C with a rotary vacuum evaporator (Eppendorf AG, Germany). The concentrated samples were centrifuged at 10,000 × *g* and 4°C for 10 min, and the supernatants were filtered through a 0.22 μm syringe filter (JinTeng, China). Then, amino acids in the filtrate of each sample were derivatized. The concentrations of the derivatized DAP in the samples were determined by HPLC on an Inertsil ODS-3 column (5 μm; 250 mm × 4.6 mm) at 25°C. The mobile phase consisted of 0.03 mol L^−1^ formic acid (mobile phase A) and acetonitrile (mobile phase B). Samples (10 μL) were analyzed using a linear elution gradient from 27% to 47% acetonitrile with a flow rate of 1.5 mL min^−1^. DAP was detected at 340 nm, and the run time was 30 min.

### Enrichment cultivation and diversity analysis of DAP-utilizing bacteria.

The enrichment medium contained 3% sea salt (Sigma, USA), 5 mM DAP (Sigma, USA), and 0.2 M Tris-HCl buffer (pH 8.0). To recover DAP-utilizing bacteria from the samples (Table S1), 0.2 mL from each sample was inoculated into 20 mL of the enrichment medium in 50 mL conical flasks, which were incubated at a 20°C shaking incubator for 4 days at 180 rpm. Afterward, 0.2 mL of each enrichment culture was inoculated into 20 mL fresh enrichment medium and incubated under the same conditions; this was repeated three times to enrich the DAP-utilizing bacteria. Total DNA of the recovered DAP-utilizing bacteria was extracted with a PowerWater DNA isolation kit (MOBIO, USA), and the bacterial 16S rRNA genes were amplified by the PCR program shown in Table S3 with EasyTaq DNA polymerase (Trans, China) and barcoded-tag primers (Table S4) (43). The amplified bacterial 16S rRNA genes were sequenced (1.2 to 1.8 kb) using a PacBio RSII instrument at the Genomics Institute in Shenzhen, China. Primers and adapter were removed by PacBio (primer_align: -d 1500 -l 1200 -u 1800 -m 2 -r). Reads were filtered by SMRT (read quality, <0.99) (44). Chimeric sequences were removed using UCHIME in the software package USEARCH (45). The sequences were merged, filtered, pooled, dereplicated, and finally assigned to each operational taxonomic unit (OTU) with 97% identity using mothur (v1.39.5) (46). For taxonomical assignment, the representative OTU sequences were compared with the 16S rRNA gene database NCBI 2017 at an 80% confidence threshold by the Bayesian last common ancestor algorithm (BLCA) (47). The number of total reads and OTUs is shown in Table S5.

### Isolation and identification of DAP-utilizing bacteria.

To isolate DAP-utilizing bacterial strains, the enrichment cultures were diluted to six concentrations (10^−1^, 10^−2^, 10^−3^, 10^−4^, 10^−5^, and 10^−6^), and then the diluted cultures were spread onto screening plates containing Ndap solid medium, composed of 5 mM DAP, 5 mM glucose, 3% sea salt, 1.5% agar, and 0.2 M Tris-HCl buffer (pH 8.0). The plates were incubated at 20°C for 3 to 7 days until detectable colonies formed. Morphologically distinct colonies were selected, transferred into 5 mL of Ndap liquid medium, and incubated at 20°C with shaking at 180 rpm for 4 days. The resulting cultures were spread onto the screening plates and incubated at 20°C until visible colonies formed. Selected colonies were purified by three consecutive transfers in the selective medium.

The purified isolates were cultured in Difco marine broth 2216 (2216E) medium (Becton, Dickinson and Company, USA) at 25°C and 180 rpm to an OD_600_ of 0.8. Genomic DNA of each isolate was extracted with a bacterial genome DNA extraction kit (BioTeke, China). The 16S rRNA genes of the isolates were amplified by the PCR program shown in Table S3 with EasyTaq DNA polymerase (Trans, China) and the primers 27F and 1492R (Table S4). The amplified genes were ligated into the pMD-19T cloning vector (TaKaRa, Japan), which was then transformed into Escherichia coli DH5α. The transformants containing the recombinant vectors were sent to Beijing Genomics Institute (China) for 16S rRNA gene sequencing. Isolates with one or more different bases in their 16S rRNA gene sequences were considered different strains. The 16S rRNA gene sequence of each strain was searched with BLASTn against the GenBank database to detect the top-hit strain as well as the sequence identity between the query and subject sequences. Accession numbers for the 16S rRNA gene sequences of the isolated strains in NCBI’s GenBank are shown in Table S6.

The ability of the isolated strains to utilize DAP was analyzed by detecting their growth at 25°C and 180 rpm in the Ndap liquid medium. The same medium without DAP and the same medium with Lys instead of DAP were used as controls.

### Genome sequencing and analysis.

Strains for genome sequencing were cultured under the conditions described above for the purified isolates. When the optical density at 600 nm (OD_600_) of the culture reached 0.8, the cells in the culture were collected by centrifugation at 10,000 × *g* for 10 min at 4°C. Genomic DNA was extracted from the cells with an E.Z.N.A. bacterial DNA kit (Omega, USA), and 2 μg of DNA (OD_260/280_ = 1.8 to 2.0; >50 ng/μL) was used to construct libraries. Bacterial genomes were sequenced and assembled using the PacBio third-generation sequencing platform at the Tianjin Biochip Corporation (China) (48). Genome *de novo* assembly (read length > 200; read quality > 80) was performed using HGAP 4 (http://www.pacbiodevnet.com/HGAP) of PacBio SMRT Link (V6.0.0.47841) (49, 50). The genome was annotated using the RAST annotation pipeline (51). The *lysA* genes were detected based on the gene annotation and were further confirmed by a BLASTp search against the GenBank database (sequence identity > 30%; sequence alignment coverage > 50%).

### Transcriptomic analysis.

The culture conditions of *Thalassospira* sp. strain A40-3 were the same as those used for genome sequencing. The cells in the culture were collected by centrifugation at 4,000 × *g* for 10 min at 4°C and washed three times with 3% sea salt solution. The cells were rested for 2 h at 4°C and then divided into three. One part was taken as the initial-phase (0-h) sample. The other two were separately inoculated into Ndap medium and the same medium with Lys instead of DAP and then cultured at 25°C and 180 rpm. Samples were taken from the cultures at the middle of the logarithmic growth phase and named DAP and Lys, respectively. The 0-h, DAP, and Lys samples were prepared in three replicates. The cells in each sample were collected by centrifugation at 4,000 × *g* and 4°C for 10 min. The resulting pellets were frozen in liquid nitrogen and sent to Beijing Agriculture Biotechnology Research Center (China) for transcriptomic sequencing and analysis. Total RNA was extracted from the cells using the TRIzol reagent according the manufacturer’s instructions (Invitrogen, USA). Genomic DNA was removed using DNase I (TaKaRa, Japan), and rRNA was removed with a RiboZero rRNA removal kit (Epicenter, USA). RNA quality was determined using a 2100 Bioanalyzer (Agilent, USA) and quantified using an ND-2000 instrument (NanoDrop Technologies, USA). High-quality RNA samples (5 μg; OD_260/280_ = 1.8 to 2.0) were used to construct the sequencing libraries. The cDNA libraries of the samples were sequenced using an Illumina HiSeq 2000 instrument with a read length of 90 bp. The raw paired-end reads were trimmed and quality controlled by Trimmomatic with the parameters SLIDINGWINDOW:4:15 and MINLEN: 75 (52). After clean sequencing data were obtained, the reads were mapped to the genome of *Thalassospira* sp. strain A40-3 with SOAP2 (53). The read counts were then progressed with the edgeR package (54) for normalization and differential expression analysis. Significantly differentially expressed genes were identified by a false-discovery-rate (FDR)-adjusted *P* value of <0.5 and a fold change value higher than 2. *P* values were calculated by the method of Fisher’s exact test with FDR correction.

### Real-time qPCR analysis.

The culture conditions of the bacterial strains (A40-3, A40-4, B30-1, and B31-7) were the same as those described for A40-3 in “Transcriptomic analysis.” Bacterial cells were sampled at the initial phase (0 h) and the early and middle logarithmic growth phases in each of the three replicates. Total RNA was extracted using the RNeasy minikit (Qiagen, USA). Then, the RNA quality was determined as described in “Transcriptomic analysis.” The extracted RNA (200 ng) was subsequently reverse-transcribed using the TransScript all-in-one first-strand cDNA synthesis super mix for qPCR (Trans, China), and then qPCR was performed using a LightCycler II 480 system (Roche, Switzerland) following the instructions for SYBR Premix Ex Taq (TaKaRa, Japan). The cycling threshold (*C_T_*) values were calculated by using the LightCycler 480 software. The relative expression level was indicated as fold change, which was calculated using the LightCycler 480 software according to the 2^−ΔΔ^*^CT^* method using the gene *recA* and the 0-h sample for normalization (55). The *recA* gene was used as the reference gene. The primers used in this experiment are listed in Table S4.

### Mutant construction.

The gene knockout (Δ*lysA*) mutant of strain *Halomonas* sp. strain A40-4, as well as the complemented (Δ*lysA*/pBB*lysA*) mutant strain, were constructed as described previously (56, 57). A 1,058 bp upstream homologous fragment was amplified from the region upstream of *lysA* of A40-4 using the primers A40-4-1F and A40-4-1R. A 1,059 bp downstream homologous fragment was amplified from the region downstream of *lysA* of A40-4 using the primers A40-4-2F and A40-4-2R. These two DNA fragments were fused into a 2,117 bp fragment by overlap PCR and introduced into plasmid pK18*mobsacB*-Ery (58). The constructed suicide vector for *lysA* knockout was named pK18-*lysA* and was transferred into *Halomonas* sp. strain A40-4 by conjugal transfer using E. coli WM3064. A 1,272 bp complementary *lysA* gene fragment was amplified from A40-4 *lysA* using primers pB4-F and pB4-R and was introduced into plasmid pBBR1MCS-2 (59) to construct the complementary plasmid pBB*lysA*, which was then transferred into the Δ*lysA* mutant by conjugal transfer using E. coli WM3064 to construct the complemented Δ*lysA*/pBB*lysA* strain. All mutations were verified by DNA sequencing. The primers and plasmids used in this experiment are listed in Tables S3 and S7. The growth (OD_600_) of A40-4 and the Δ*lysA* and Δ*lysA*/pBB*lysA* strains in Ndap medium at 25°C and 180 rpm was monitored to analyze their ability to utilize DAP as a sole N source. The primers and plasmids used in this experiment are listed in Tables S4 and S7, respectively.

### Gene cloning and expression and purification of DAP decarboxylase.

The *lysA* nucleotide sequences were amplified from *Halomonas* sp. strain A40-4, *Thalassospira* sp. strain A40-3, *Alteromonas* sp. strain B31-7, and *Thalassospira* sp. strain B30-1 by the PCR program shown in Table S3 with FastPfu DNA polymerase (Trans, China) and the primers shown in Table S4 and then introduced into a pET22b expression plasmid with an In-Fusion HD cloning kit (TaKaRa, Japan). The constructed recombinant plasmids were transformed into E. coli BL21(DE3). Transformants were cultured at 37°C and 180 rpm in LB liquid medium containing 100 mg mL^−1^ ampicillin. When the OD_600_ of the cultures reached approximately 0.6, 1 mM isopropyl-b-d-thiogalactopyranoside (IPTG) was added to the culture for the induction of protein expression. Then, the cultures were incubated at 15°C and 110 rpm for 14 h. After incubation, the cells in the cultures were harvested, resuspended in lysis buffer (50 mM Tris-HCl, 100 mM NaCl; pH 8.0), and disrupted by high pressure using a JN-02C low-temperature ultrahigh-pressure continuous-flow cell disrupter (JNBIO, China). The recombinant His-tagged LysA proteins in the resulting extracts were purified by Ni affinity chromatography (Qiagen, USA) and desalted with disposable PD-10 desalting columns (GE Healthcare, Sweden). Protein concentrations were determined by using the Pierce bicinchoninic acid (BCA) protein assay kit (Thermo Scientific, USA).

### Assay of enzyme activity.

DAP decarboxylase activity of the recombinant LysA proteins was assayed by the method of Peverelli and Perugini (60). The purified recombinant LysA proteins from *Alteromonas* sp. strain B31-7, *Halomonas* sp. strain A40-4, *Thalassospira* sp. strain A40-3, and *Thalassospira* sp. strain B30-1 were mixed with 200 mM Tris-HCl (pH 8.0), 100 mM pyridoxal phosphate (PLP), and 5 mM DAP. The mixtures were incubated at 25°C for 10 min, and the reactions were stopped by adding 10% trifluoroacetic acid (TFA) to the mixtures. The control assay had the same reaction system except that recombinant LysA was not added, and it was performed under the same reaction conditions. The enzymatic activity of DAP decarboxylase was determined by measuring the production of Lys using an automatic amino acid analyzer (L-8900; Hitachi, Japan). One unit of enzyme was defined as the amount of enzyme that converted DAP into 1 μmol Lys per minute.

### Investigation of the diversity and distribution of LysA-containing bacteria in the ocean.

The full-length sequence (423 amino acid residues) of the LysA protein from *Thalassospira* sp. strain A40-3 (Fig. S4) was queried in the *Tara* Oceans database (http://ocean-microbiome.embl.de/companion.html) using tBLASTn to search for *lysA*-containing bacteria, with an E-value cutoff of 1E−30 (61, 62). The taxonomic composition of the microbial community was analyzed based on the 16S rRNA gene sequences from the *Tara* Oceans database.

The distribution of *lysA*-containing bacteria was assayed by the method of Carrión et al. (63) and analyzed and visualized with scatterpie (64), ggplot2 (65), and maps (66) in the R software (version 4.1.2). BLAST (version 2.11.0+) (67) was used to obtain the number of *lysA* hits against the local *Tara* Oceans database with an E-value cutoff of 1E−30. The numbers of unique hits of *lysA* were normalized to the number of unique *recA* sequences to estimate the abundance of *lysA* in the *Tara* Oceans database. The number of unique *recA* genes in the *Tara* Oceans database was obtained using the 120 *recA* sequences (63) as probes to perform BLASTp searches from the *Tara* Oceans database, with an E-value cutoff of 1E−30.

### Data availability.

The main data supporting the findings of this study are available within the article and the supplemental material. All data and materials supporting the findings of this study are available from the corresponding author upon reasonable request. PacBio 16S rRNA gene data have been deposited in NCBI’s Sequence Read Archive (SRA) under the project accession number PRJNA810257. The 16S rRNA gene sequences of the isolated strains have been deposited in NCBI’s GenBank with the accession numbers shown in Table S6. The whole-genome sequences of 4 strains generated in this study are publicly available in NCBI’s GenBank with accession numbers CP065428 (A40-3), CP065233 (B31-7), CP065232 (B30-1), and CP065230 (A40-4). All the RNA-seq read data have been deposited in NCBI’s SRA under the project accession number PRJNA743367.
