# Supplementary material for: Diaminopimelic Acid Metabolism by Pseudomonadota in the Ocean
Source: Microbiol Spectr. 2022 Aug 30;10(5):e00691-22. doi: 10.1128/spectrum.00691-22 (PMC9602339; doi:10.1128/spectrum.00691-22)
Supplement: Supplemental file 1 — Tables S1-S7; Fig. S1-S8. Download spectrum.00691-22-s0001.pdf, PDF file, 1.6 MB [file spectrum.00691-22-s0001.pdf]

Diaminopimelic acid metabolism by Pseudomonadota in the ocean

Running title: Bacterial metabolism of oceanic diaminopimelic acid

Li-Yuan Zheng[1], Ning-Hua Liu[1], Shuai Zhong[1], Yang Yu[1], Xi-Ying Zhang[1], Qi-Long Qin[1], Xiao-Yan Song[1], Yu-Zhong Zhang[1,2,3], Huihui Fu[2,3], Min Wang[2], Andrew McMinn[2,4], Xiu-Lan Chen[1,3]*, Ping-Yi Li[1]*

[1]State Key Laboratory of Microbial Technology, and Marine Biotechnology Research Center, Shandong University, Qingdao, 266237, China;

[2]College of Marine Life Sciences, and Frontiers Science Center for Deep Ocean Multispheres and Earth System, Ocean University of China, Qingdao, 266003, China;

[3]Laboratory for Marine Biology and Biotechnology, Pilot National Laboratory for Marine Science and Technology, Qingdao, China;

[4]Institute for Marine and Antarctic Studies, University of Tasmania, Hobart, Tasmania, Australia;

*Corresponding authors: Ping-Yi Li, lipingyipeace@sdu.edu.cn; Xiu-Lan Chen, cxl0423@sdu.edu.cn

Table S1. Information of the sampling sites. The Distance column shows the kilometers from station A4, A10 or B3 to station A1.

Station	Distance (km)	Sample	Depth (m)	Location
A1	0	A1/0	0	153.42111°E17.39111° N
		A1/2000	2000	153.42111°E17.39111° N
		A1/3000	3000	153.42111°E17.39111° N
		A1/5000	5000	153.42111°E17.39111° N
A4	20.9	A4/0	0	153.22500°E17.40361° N
		A4/1000	1000	153.22500°E17.40361° N
		A4/2000	2000	153.22500°E17.40361° N
A10	68.9	A10/0	0	152.77166°E17.39833° N
		A10/1000	1000	152.77166°E17.39833° N
		A10/2000	2000	152.77166°E17.39833° N
B3	31.5	B3/0	0	153.16027°E17.52638° N
		B3/1000	1000	153.16027°E17.52638° N
		B3/3000	3000	153.16027°E17.52638° N

Table S2. Information on LysAs of the four genome-sequenced strains.

Strain	Genome size (Mb)	*lysA* length (bp)	Identity (%)[a]	LysA length (aa)	LysA ID	Identity (%)[b]
A40-3	4.19	1272	85	423	QPL46057.1	38
A40-4	4.00	1272	65	423	QPO11377.1	38
B30-1	4.62	1263	97	421	QPL35139.1	39
B31-7	4.39	1251	68	417	QPL50280.1	40

[a] The identity of the sequence of the *lysA* gene of each strain to that of *lysA* (CP004388.1) of *Thalassospira* sp. M-5.

[b] The identity of the sequence of protein LysA of each strain to that of LysA (WP_064496723.1) of *Methanococcus jannaschii* (Ray *et al*., 2002).

Table S3. Programs and reaction mixtures used in PCRs.

Enzyme	Reaction Mixture (50 μL)	Programs		
EasyTaq® DNA Polymerase (Trans, China)	1 μL Primers (10 μM)	94 °C	5 min	
	4 μL DNTPs (2.5 mM)	94 °C	30 s	30 cycles
	5 μL 10x EasyTaq Buffer	55 °C	30 s	
	1 μL EasyTaq DNA Polymerase	72 °C	1 kb/min	
	200 ng Genome DNA	72°C	5 min	
	Add Nuclease-free water to 50 μL			
FastPfu® DNA Polymerase (Trans, China)	1 μL Primers (10 μM)	95 °C	2 min	
	4 μL DNTPs (2.5 mM)	95 °C	20 s	30 cycles
	10 μL 5x FastPfu Buffer	55 °C	20 s	
	1 μL FastPfu DNA Polymerase	72 °C	2 kb/min	
	200 ng Genome DNA	72°C	5 min	
	Add Nuclease-free water to 50 μL			

Table S4. Primers used in this study. Primers for gene amplification and plasmid or mutant construction were designed by SnapGene. Primers for qPCR were designed by Beacon Designer 7. All primers were synthesized by Tsingke Biotechnology (China).

**Primer**	**Sequence (5'-3')**	**Description**
27F	AGAGTTTGATCCTGGCTCAG	Amplification of 16S rRNA genes
1492R	GGTTACCTTGTTACGACTT	
A40-3pr-F	AAGAAGGAGATATACATATGAACCATTTTGAATATATCAACAGCGA	Amplification of *lysA* genes
A40-3pr-R	TGGTGGTGGTGGTGCTCGAGTGCGTTGCTGAGCCAGGAGG	
A40-4pr-F	AAGAAGGAGATATACATATGGATCATTTTAACGACCGTGACGG	
A40-4pr-R	TGGTGGTGGTGGTGCTCGAGGCAGCCTCCTTCGGGCAGCA	
B30-1pr-F	AAGAAGGAGATATACATATGAACCATTTTGAATATATTGATGGCG	
B30-1pr-R	TGGTGGTGGTGGTGCTCGAGTCCAGCCAGTCGGGAATGC	
B31-7pr-F	AAGAAGGAGATATACATATGGATTTTTTTGCTTATAAAAACAACC	
B31-7pr-R	TGGTGGTGGTGGTGCTCGAGCGTAAGTTTATGCTCACCTTTCC	
A404-e1F	GTAAAACGACGGCCAGTGCCAAGCTTGTGGTGAAGTGCTTGCCATG	Construction of Δ*murE*

A404-e1R	ACGTGACCGAGGGTCCAGTCCATCTAGGCAATCTCCTTCCCACTAGTC	
A404-e2F	ATAGAGAGGCGGACTAGTGGGAAGGAGATTGCCTAGATGGACTGGACCCTCG	
A404-e2R	GTCATAAGATTAGTCACTGGGGATCCCAGACGCTCGATTACCTGCG	
A404-f1F	GTAAAACGACGGCCAGTGCCAAGCTTCAATTTGAGCCGTGATCATC	Construction of Δ*murF*
A404-f1R	CGCCGACATGAGCACCGGTCACATTGGTAATAACCTAGAGCGCCTCCCGAGCGG	
A404-f2F	CAGTCCGCACTGGCCGCTCGGGAGGCGCTCTAGGTTATTACCAATGTGACCG	
A404-f2R	GTCATAAGATTAGTCACTGGGGATCCTAACATGAACGGCGTTTACC	
A40-4-1F	GTAAAACGACGGCCAGTGCCAAGCTTACTTGCTGGCCTGGCAAGAG	Construction of Δ*lysA*
A40-4-1R	CATAAAGTCGTTGCCGAGGCCGTGCATTTTGGTGAAGTGCCCTGCTGATCGACATTATCG	
A40-4-2F	CCGCTCTACCTGCCCGATGACGATAATGTCGATCAGCAGGGCACTTCACCAAAATGCACG	
A40-4-2R	GTCATAAGATTAGTCACTGGGGATCCCAACTGCTGCAGCAGCCCTTC	
pB4-F	TCGACGGTATCGATAAGCTTGATGGATCATTTTAACGACCGTG	Construction of complementary plasmid pBB-*lysA*
pB4-R	CTCTAGAACTAGTGGATCCCCTTAGCAGCCTCCTTCGGGC	
40-3 ly-F	AAACGCTTTGTCATTCTT	Quantitative PCR of gene *lysA*
40-3 ly-R	GGATGATTTCGTGATAGG	
40-3F-F	AACCTTTACCCTGATTGAT	Quantitative PCR of gene *murF*
40-3F-R	TGTGACAGGACCTTAATC	
40-3E-F	GGCATTGGCATTCTTCAG	Quantitative PCR of gene *murE*
40-3E-R	GACCGTATCACCGACAAC	
40-3rec-F	TGAAGATTGGTGTGATGT	Quantitative PCR of gene *recA*
40-3rec-R	GGACAGAGGCATAGAATT	
40-4 ly-F	ATTGGAGTTTGCCTTTAC	Quantitative PCR of gene *lysA*
40-4 ly-R	GCCTTGTTATGTCTATTCA	
40-4F-F	CGCATTACTCACATTATG	Quantitative PCR of gene *murF*
40-4F-R	TTCACGCTTAACTATCAA	
40-4E-F	CATACTGCCGTGATAATC	Quantitative PCR of gene *murE*
40-4E-R	GCTGGATAGCGTTGATAT	

40-4rec-F	TTAAAGTGGTCAAGAACAAG	Quantitative PCR of gene *recA*
40-4rec-R	TATATGCCTTTGCCGTAG	
30-1 ly-F	TATATTGATGGCGTCCTT	Quantitative PCR of gene *lysA*
30-1 ly-R	GGTGGAATAGCAATAGAAC	
30-1F-F	GATTGACGATGCCTATAACG	Quantitative PCR of gene *murF*
30-1F-R	ATCCTTCGGGTTTGCTTT	
30-1E-F	AACGCAACGATATTCTTAT	Quantitative PCR of gene *murE*
30-1E-R	CATCATCAAAAGGCAGAA	
30-1rec-F	ATGAAGATCGGTGTGATG	Quantitative PCR of gene *recA*
30-1rec-R	GGACAGACGCATAGAATT	
31-7 ly-F	GCATCAACATCTGGATTA	Quantitative PCR of gene *lysA*
31-7 ly-R	GTCTGAACCTGAATTACAT	
31-7F-F	CTAGACGATACCTACAAC	Quantitative PCR of gene *murF*
31-7F-R	ATGATAATAGCGAGACTT	
31-7E-F	TGTTATACAGGCGGTTAA	Quantitative PCR of gene *murE*
31-7E-R	GGCATAATCCACAATCAC	
31-7rec-F	CCTATACCTAGAGCGATA	Quantitative PCR of gene *recA*
31-7rec-R	TGGACGATAACAAATCAA	

Table S5. 16S rDNA sequencing results for diversity analysis of DAP-utilizing bacteria.

samples	Total reads	Total OTUs
A4/0	13680	150
A4/1000	12618	65
A4/2000	11440	50
B3/0	14207	51
B3/1000	12315	51
B3/3000	13068	49
A1/0	13508	98
A1/2000	10824	122
A1/3000	18574	89
A1/5000	15747	110
A10/0	14144	108
A10/1000	13834	90
A10/2000	24914	87

Table S6. The accession numbers for the 16S rDNA sequences of the isolated strains

Strain	Accession number	Strain	Accession number
Erythrobacter sp. A30-3	ON600590	*Pseudooceanicola* sp. B31-1	ON619587
Sulfitobacter sp. B30-2	ON600591	*Thalassospira* sp. B30-3	ON619588
Thalassospira sp. A40-3	ON600592	*Labrenzia* sp. A42-5	ON619589
Halomonas sp. A40-4	ON600593	*Alteromonas* sp. A41-4	ON619590
Thalassospira sp. B30-1	ON600594	Zunongwangia sp. B30-5	ON619591
Alteromonas sp. B31-7	ON600595	Thalassospira sp. B31-4	ON619592
Microbacterium sp. A32-1	ON600596	Pseudoalteromonas sp. A41-2	ON619595

Table S7. Plasmids and strains used in this study.

Plasmids/Strains	Description	Source or reference
pMD19T	Used for 16S rRNA genes sequencing	TAKARA, Japan
pK18*mobsacB*-Ery	Used for gene knockout	Wang *et al*., 2015
pK18-*lysA*	Construction of Δ*lysA*	This study
pBBR1MCS-2	Used for gene expression	Kovach *et al*., 1995
pBBR-*lysA*	Construction of complementary strains Δ*lysA*/pBB-*lysA*	This study
pET22b	Used for gene expression	Norvagen, Germany
pET-*lysA*$^{A40-3}$	Expression of DAPDC^{A40-3} protein	This study
pET-*lysA*$^{A40-4}$	Expression of DAPDC^{A40-4} protein	This study
pET-*lysA*$^{B30-1}$	Expression of DAPDC^{B30-1} protein	This study
pET-*lysA*$^{B31-7}$	Expression of DAPDC^{B31-7} protein	This study
Escherichia coli DH5α	Used for plasmid amplification	Tsingke Biotechnology, China
E. coli WM3064	Used for plasmid transfer	Wang *et al*., 2015
E. coli BL21	Used for protein expression	Vazyme, China

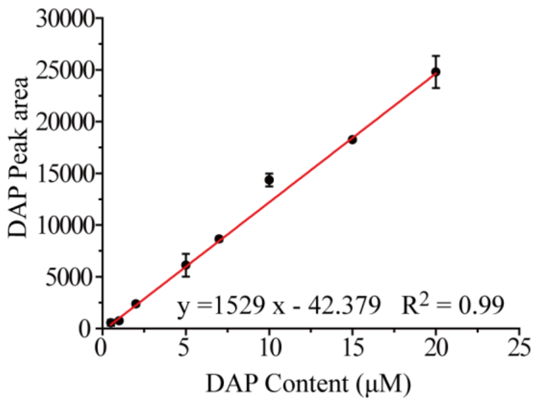

Figure S1. The standard curve of DAP detected by FDAA derived HPLC. The standard curve of DAP was determined with 0 to 25 μM DAP. The graph shows mean values of triplicate experiments.

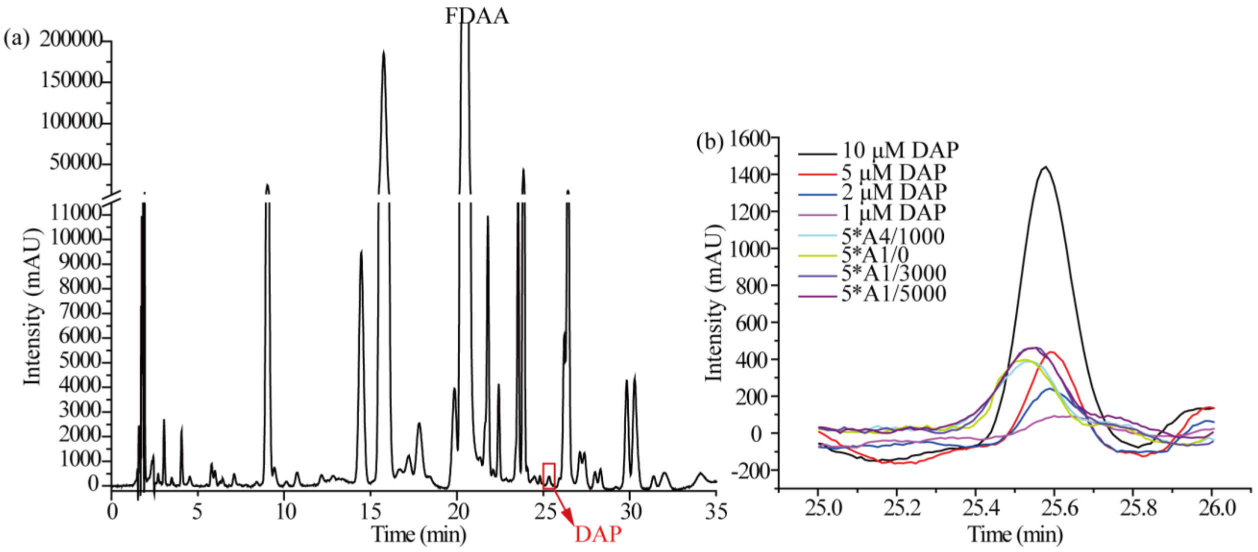

Figure S2. The raw HPLC chromatograms from a sample and standard. (a) The raw HPLC chromatograms of A1/0 seawater sample. The sample was concentrated (1:5) before analysis. FDAA was detected at 20.2 min. **(b)** The HPLC chromatograms of DAP standards and concentrated samples from A4/1000, A1/0, A1/3000 and A1/5000.

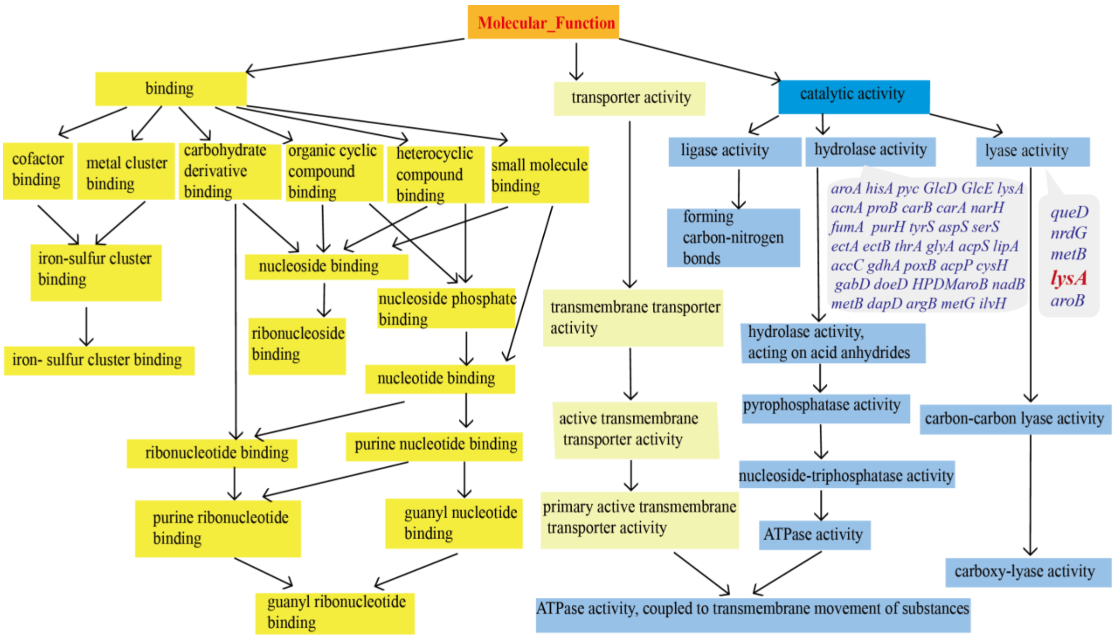

Figure S3. Enriched GO (gene ontology) terms of up-regulated genes in strain *Thalassospira* sp. A40-3 cultured with DAP as the sole nitrogen source to the middle of the logarithmic growth phase compared with the 0 h samples taken at the initial phase.

```
ATGAACCATTTTGAATATATCAACAGCGAACTTTACGCCGAAGGCGTCTCGATCCGGGAAATGGCTGACAAGGTCGGAACCCCGTTCTTCTGCTAT    96
M N H F E Y I N S E L Y A E G V S I R E M A D K V G T P F F C Y

TCGACCGCGACACTGGAACGCCATTACAAGGTCTATGCCGACGCATTTGACGGCCTTGATGCCACCGTGTGCTTTGCCGTTAAGGCAAACTCCAAT    192
S T A T L E R H Y K V Y A D A F D G L D A T V C F A V K A N S N

CAGGCAGTCCTGAAAACACTCGCCAATCTTGGTGCGGGTGCGGACGTTGTTTCGGTTGGCGAAATGCGCCGTGCGTTGCGTGCGGGCATGCCCCCG    288
Q A V L K T L A N L G A G A D V V S V G E M R R A L R A G M P P

GCAAAGATCATCTTTTCCGGGGTTGGCAAGGCCGATGCCGAAATGCGCGAAGCCCTTGACGCCGACATTGCCCAGATCAATGTCGAAAGCATCCCC    384
A K I I F S G V G K A D A E M R E A L D A D I A Q I N V E S I P

GAACTGGTTGAGCTGAACCGTGTTGCCATCGACATGGGCAAAAAGGCGCGCATTGCGCTGCGCGTCAACCCGCATGTCGATGCCAAAACCCACGAA    480
E L V E L N R V A I D M G K K A R I A L R V N P H V D A K T H E

AAGATCGCGACCGGCAAGGCTGAAAACAAATTCGGCATCGACTGGACCCGCGCGATCGAGGTTTACCGCGAAGCCGCCGCCATGGACGGCATCGAG    576
K I A T G K A E N K F G I D W T R A I E V Y R E A A A M D G I E

GTTACCGGGATCGCCATGCATATCGGCTCCCAGTTGACCGACCTTGCCCCCTTCCGCGAGGCATTTACACGTCTGGCCGGTCTGGTTGAACAGTTG    672
V T G I A M H I G S Q L T D L A P F R E A F T R L A G L V E Q L

CGATCTGAGGGCATCGATATCCGCAATCTTGATCTCGGTGGCGGGCTTGGCATTGCCTATCAGGGTGAAACGCCGCCGCTTCCCGATGCCTATGGT    768
R S E G I D I R N L D L G G G L G I A Y Q G E T P P L P D A Y G

CAGATGGTGCGCGAAACCGTCGGCCATCTTGGCTGCCACATCACGCTGGAACCGGGCCGTTTGATTGCCGGCAATGCCGGGATCATGGTATCGCGC    864
Q M V R E T V G H L G C H I T L E P G R L I A G N A G I M V S R

GTCATGTATATCAAAGATGGCGAAGCCAAACGCTTTGTCATTCTTGATGCCGCGATGAACGACCTGATCCGCCCGACACTGTATGGCGCCTATCAC    960
V M Y I K D G E A K R F V I L D A A M N D L I R P T L Y G A Y H

GAAATCATCCCGGTCGATGAACAGACTGACGGTGACAAGCAGGTCAAGGTTGATGTGGTCGGCCCGGTTTGCGAAACCGGTGACACATTTGGCAAG    1056
E I I P V D E Q T D G D K Q V K V D V V G P V C E T G D T F G K

GACCGCGACCTTCCTGATGACCTAAAGGCCGGTGATCTGGTCGCTGTCATGTCGGCGGGGGCCTATGGCGCGGTCATGTCATCGACCTACAACACC    1152
D R D L P D D L K A G D L V A V M S A G A Y G A V M S S T Y N T

CGTGCGCTGACACCCGAAGTACTGGTCAAGGGCAACAACTTTGCCATCGTCCGCCCGCGTCAGGAAATTGACGATCTGATCAATATGGATCTGGTC    1248
R A L T P E V L V K G N N F A I V R P R Q E I D D L I N M D L V

CCCTCCTGGCTCAGCAACGCATAA
P S W L S N A *
```

Figure S4. The Nucleotide sequence of gene *lysA* and the deduced amino acid sequence of LysA of *Thalassospira* sp. A40-3.

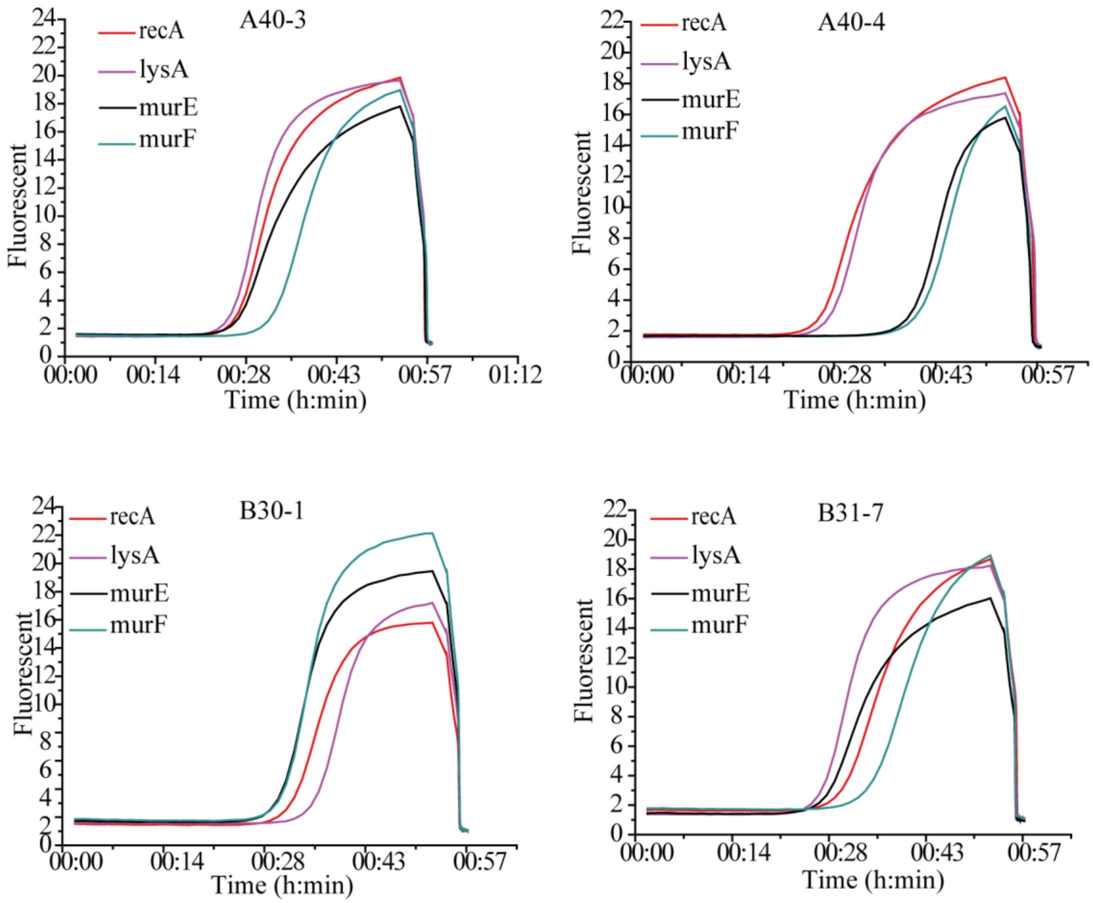

Figure S5. Representative qPCR amplification curves of genes *recA*, *lysA*, *murE* and *murF* in strains A40-3, A40-4, B30-1 and B31-7.

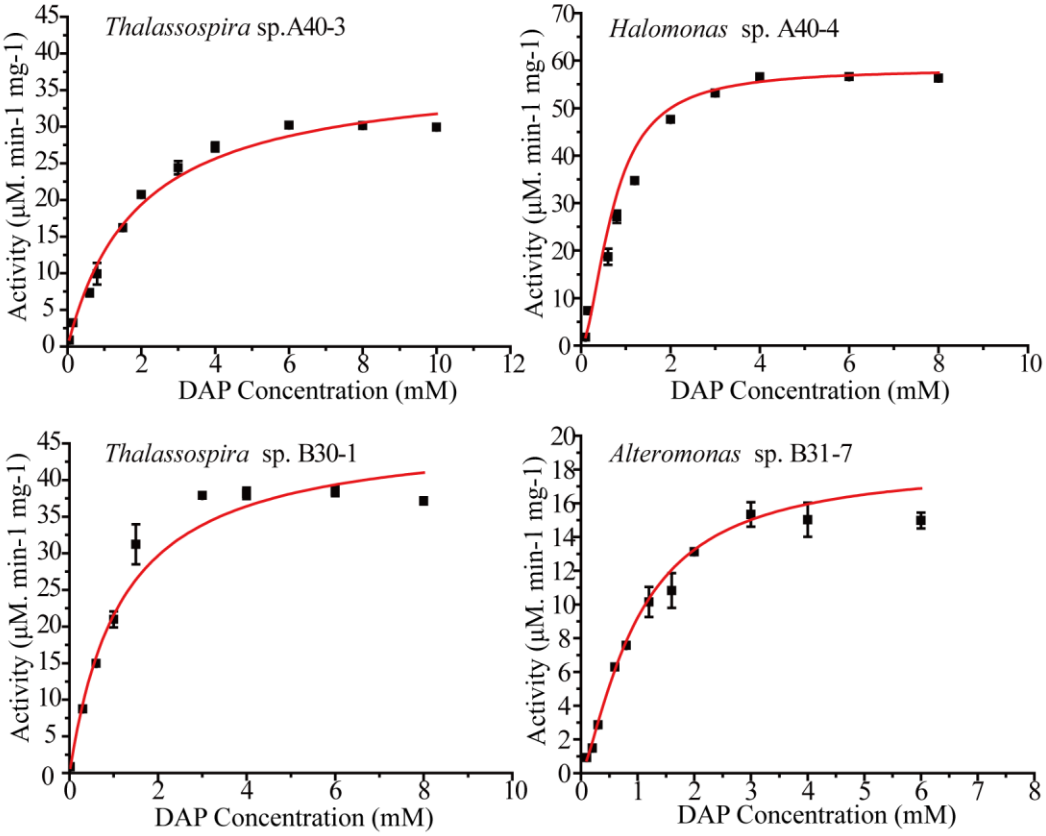

Figure S6. Non-linear fit curves for the decarboxylation of DAP by the LysA proteins. These assays were carried out in 0.2 M Tris-HCl containing 0.1 M Pyridoxal Phosphate and different concentration of DAP at 25°C. Kinetic parameters were calculated by non-linear regression fit directly to the Michaelis-Menten equation using Origin8 software. The error bars represent standard deviations from triplicate experiments.

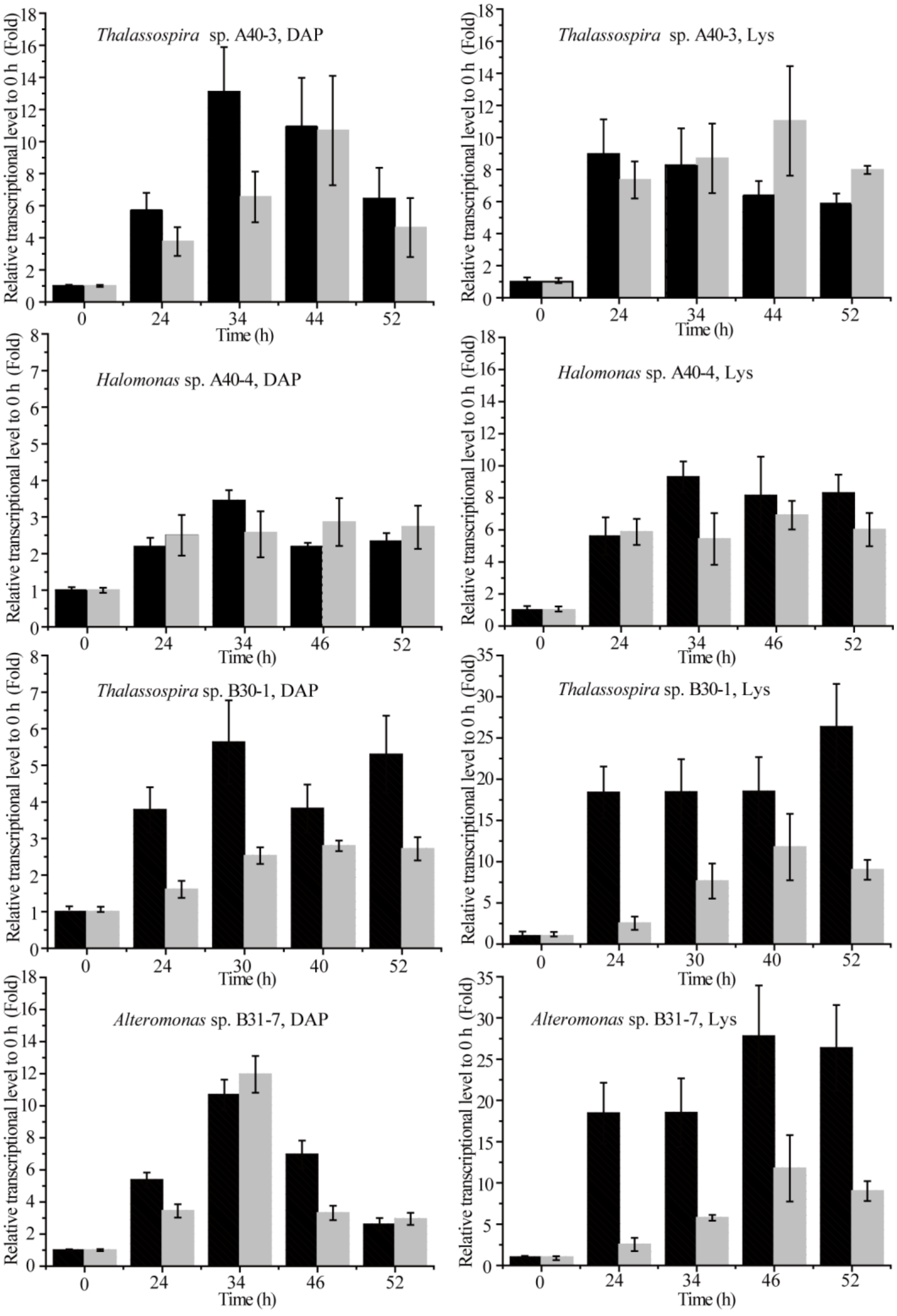

Figure S7. Relative transcriptional levels of genes *murE* and *murF* in strains B30-1, B31-7, A40-3 and A40-4. Bacteria were cultured with DAP or Lys as the sole nitrogen source at 25°C and 180 rpm. Relative

transcriptional levels of genes *murE* and *murF* were marked with black and gray, respectively. The error bars represent standard deviations from triplicate experiments.

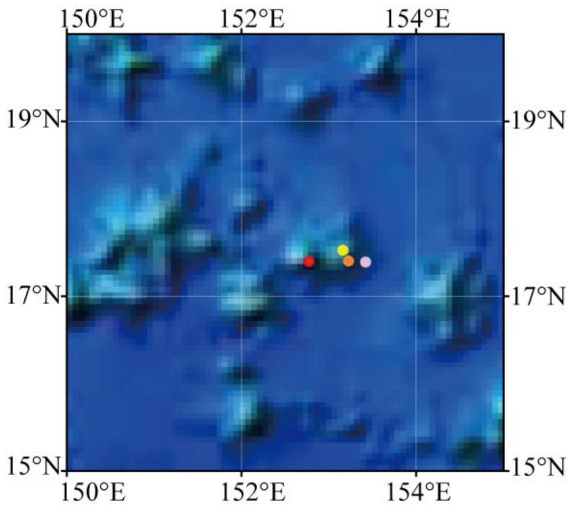

Figure S8. Location of the sampling stations. Stations A1 A4, A10 and B3 are marked in pink, orange, red and yellow, respectively.
